# Atezolizumab in combination with bevacizumab enhances antigen-specific T-cell migration in metastatic renal cell carcinoma

**DOI:** 10.1038/ncomms12624

**Published:** 2016-08-30

**Authors:** Jeffrey J. Wallin, Johanna C. Bendell, Roel Funke, Mario Sznol, Konstanty Korski, Suzanne Jones, Genevive Hernandez, James Mier, Xian He, F. Stephen Hodi, Mitchell Denker, Vincent Leveque, Marta Cañamero, Galina Babitski, Hartmut Koeppen, James Ziai, Neeraj Sharma, Fabien Gaire, Daniel S. Chen, Daniel Waterkamp, Priti S. Hegde, David F. McDermott

**Affiliations:** 1 Genentech, Inc., 1 DNA Way, South San Francisco, California 94080, USA; 2 GI Oncology Research, Drug Development Unit, Sarah Cannon Research Institute, 250 25th Avenue North, Suite 100, Nashville, Tennessee, 37203, USA; 3 Department of Internal Medicine and Melanoma Unit, Yale Cancer Center, New Haven, Connecticut 06511, USA; 4 Roche Diagnostics GmbH, Nonnenwald 2, 82377 Penzberg, Germany; 5 Beth Israel Deaconess Medical Center, 330 Brookline Avenue, Boston, Massachusets 02215, USA; 6 Dana-Farber/Brigham and Women's Cancer Center, 450 Brookline Avenue, Boston, Massachusets 02215, USA

## Abstract

Anti-tumour immune activation by checkpoint inhibitors leads to durable responses in a variety of cancers, but combination approaches are required to extend this benefit beyond a subset of patients. In preclinical models tumour-derived VEGF limits immune cell activity while anti-VEGF augments intra-tumoral T-cell infiltration, potentially through vascular normalization and endothelial cell activation. This study investigates how VEGF blockade with bevacizumab could potentiate PD-L1 checkpoint inhibition with atezolizumab in mRCC. Tissue collections are before treatment, after bevacizumab and after the addition of atezolizumab. We discover that intra-tumoral CD8^+^ T cells increase following combination treatment. A related increase is found in intra-tumoral MHC-I, Th1 and T-effector markers, and chemokines, most notably CX3CL1 (fractalkine). We also discover that the fractalkine receptor increases on peripheral CD8^+^ T cells with treatment. Furthermore, trafficking lymphocyte increases are observed in tumors following bevacizumab and combination treatment. These data suggest that the anti-VEGF and anti-PD-L1 combination improves antigen-specific T-cell migration.

## Abstract (web summary)

Cancer immunotherapy can be used in combination with other therapies for a better response. Here, the authors conduct a phase Ib clinical study and report the clinical activity and the immune response of the anti-PDL1 agent, atezolizumab, in combination with bevacizumab in ten patients with metastatic renal cell carcinoma.

## Graphical abstract

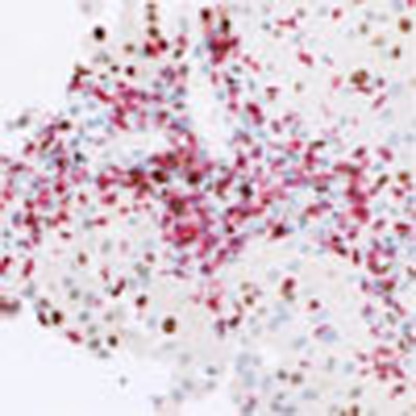

Programmed death-ligand 1 (PD-L1) is expressed on T cells and antigen-presenting cells, including dendritic cells, macrophages and tumour cells^1^. The binding of PD-L1 to the receptor programmed death-1 (PD-1) plays a central role in T-cell tolerance by inhibiting naive and effector T-cell responses^2^. Clinical experience with checkpoint inhibitors has shown that tumours co-opt the PD-L1/PD-1 signalling pathway as one key mechanism to evade immune destruction. Atezolizumab is an engineered humanized monoclonal anti-PD-L1 antibody that specifically inhibits PD-L1/PD-1 signalling to restore tumour-specific T-cell immunity^3^,^4^. It also induces durable antitumour effects for some cancer patients, including those with metastatic renal cell carcinoma (mRCC)^1^,^5^.

Vascular endothelial growth factor A (VEGF) is a secreted factor that specifically acts on endothelial cells to stimulate angiogenesis making it a critical therapeutic target in cancers such as mRCC^6^. VEGF has also been shown to exert immunosuppressive function and there is a long-established role for immunotherapy approaches such as interferon-α in mRCC^7^. The combination of interferon-α plus bevacizumab (anti-VEGF) has been evaluated in mRCC and resulted in significant improvement in progression-free survival compared with interferon-α alone leading to the approval of this combination for treatment of mRCC in the front-line setting^8^. These findings suggest that combining immunotherapy with bevacizumab can potentially augment the antitumour immune response in mRCC.

The combination of the checkpoint inhibitor ipilimumab (anti-CTLA4) and bevacizumab has been investigated recently in metastatic melanoma^9^. The study revealed extensive morphological changes in CD31^+^ endothelial cells and widespread infiltration of immune cells following combination treatment. Immune infiltrates contained significant numbers of CD8^+^ and CD163^+^ macrophages compared with ipilimumab treatment alone. Further investigation of endothelial cell alterations indicated that the treatments were adapting vessels for effective lymphocyte trafficking. While combinations of agents that inhibit the PD-1 and VEGF signalling pathways have entered clinical trials, the potential pharmacodynamic effects of this approach remain poorly understood. Here we show the potential mechanisms of action underlying the activity of bevacizumab and the combination of atezolizumab and bevacizumab in mRCC. We identify changes in antitumour immune markers that are associated with treatment.

## Results

### Study design and clinical results

**Figure 1:**
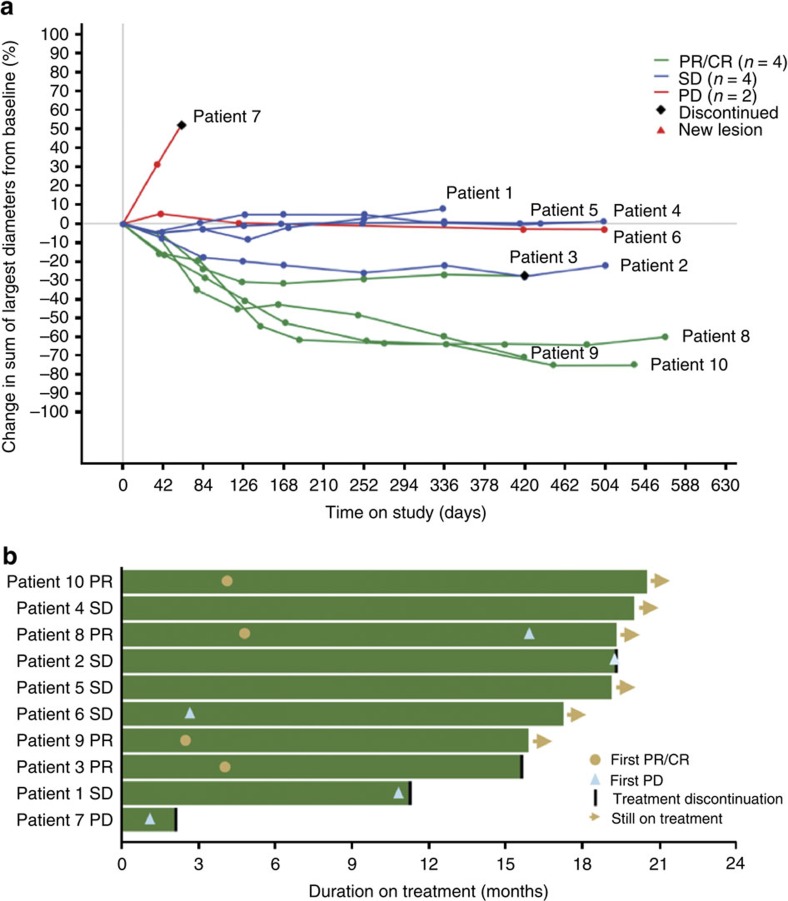
Antitumour activity of atezolizumab and bevacizumab combination. (**a**) Tumour burden over time in RCC patients. Plot of patients with RCC measuring the maximum reduction from baseline in the sum of the longest diameter for target lesions. CR, complete response; PD, progressive disease; PR, partial response; SD, stable disease. (**b**) Duration of study treatment for patients with RCC.

In this study, 10 patients with previously untreated mRCC received a single dose of bevacizumab on C1D1, followed by combined administration of atezolizumab and bevacizumab every 3 weeks beginning on C2D1 (Supplementary Table 1). The treatments were well tolerated (adverse events; Supplementary Tables 2 and 3). None of the six treatment-related grade 3–4 adverse events were deemed related to atezolizumab by study investigators^10^. Partial responses were observed in 4 of 10 patients using RECIST v1.1, while an additional 4 patients had prolonged stable disease (Fig. 1). One patient classified with progressive disease due to the appearance of a new lesion early in their treatment remains on study after almost 18 months with stable overall tumour burden. The clinical activity observed in this small cohort was higher than previously obtained with either monotherapy^5^,^11^. The median duration of response has not been reached and the median time to response was 4.2 months.

### Bevacizumab increases biomarkers of antitumour immunity

In addition to safety, tolerability and clinical activity, one key objective of this study was to evaluate mechanisms of combination activity. The trial design included a run-in period with bevacizumab to specifically interrogate the effects of bevacizumab on the local tumour immune microenvironment, followed by combination therapy with immune checkpoint blockade. Tumour biopsies and blood were collected before treatment, 15–18 days following bevacizumab and 4–6 weeks after initiation of atezolizumab and bevacizumab combination treatment.

**Figure 2:**
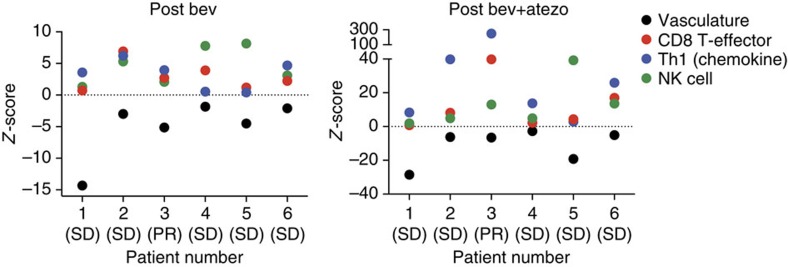
Gene expression tumour biomarkers following treatments. Levels of on-treatment tumour samples are shown relative to the baseline levels (dotted line) for the patients where the pre-treatment, post-bevacizumab (bev; blue circles) and post-combination biopsies were collected. Vascular signature genes (*ANGPT2*, *CD34*, *DLL4*, *EGFL7* and *ESM1*) are shown in black, CD8 T effector genes (*CD8A*, *CD8B*, *EOMES*, *GZMA*, *GZMB*, *IFNG* and *PRF1*) are shown in red, Th1 chemokines (*CXCL10*, *CXCL11*, *CXCL13* and *CXCL9*) are shown in blue and natural killer (NK) cell genes (*GZMB*, *KLRK1* and *SLAMF7*) are shown in green. atezo, atezolizumab.

To identify tumour markers associated with bevacizumab monotherapy or combination therapy, we used gene expression analysis using both a 90-gene-PCR-based Fluidigm panel and an 800-gene-custom Nanostring panel. Genes associated with the neo-vasculature, which reflect VEGF downstream signalling activity were significantly decreased at both on-treatment time points in all patients (Fig. 2), confirming anti-angiogenic activity of bevacizumab^12^. Conversely, there was a trend towards increased gene signatures associated with T-helper 1 (Th1) chemokines, CD8 T-effector, as well as natural killer cells (Fig. 2). In this study, bevacizumab treatment resulted in four of the six patients showing a significant increase in gene signatures related to Th1 signalling. Importantly, at the individual patient level, these signatures were delinked from the degree of reduction of the VEGF-dependent signature. FasL expression by immunohistochemistry (IHC) has been described as a potential barrier to immune cells in several cancers, including renal cell carcinoma (RCC)^13^. FASL protein expression was not measured in this study and we did not observe consistent changes in FasL gene expression with bevacizumab or combination treatment (Supplementary Fig. 1). Overall, these differences suggest bevacizumab treatment alone results in modulation of tumour immune microenvironment with Th1-related signatures reflecting the most significant treatment-induced alterations in the tumour microenvironment.

### Increases in immune cell markers are observed on-treatment

**Figure 3:**
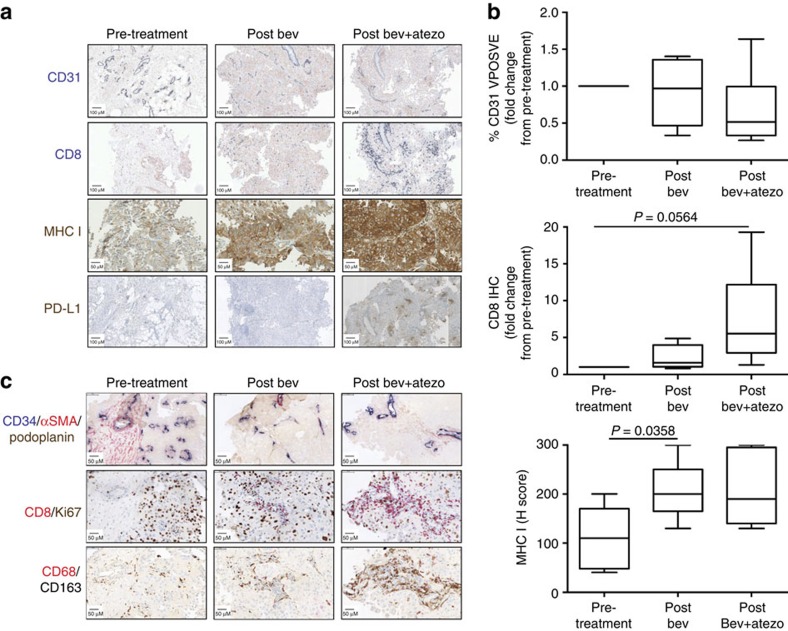
Protein expression of immune and vasculature markers in baseline and on-treatment tumour samples. (**a**) Representative images of CD8 (blue), CD31 (blue), PD-L1 (brown) and MHC-I (brown) by IHC from patient 3 tumours. (**b**) Quantitation of CD31, CD8 and MHC-I IHC. *P* values were determined by paired *t*-test. The line in the middle of the box is plotted at the median. Lines above and below the boxes represent variability outside the upper and lower quartiles. (**c**) IHC images for the indicated triple and double stains of serial sections from patient 3 tumours. A scale bar for each image representing 50 or 100 μm is shown. atezo, atezolizumab; bev, bevacizumab.

To confirm the immune and vascular gene expression changes observed in the tumour, IHC was used on pre-treatment and on-treatment tissue. Consistent with gene expression, a decrease in CD31, a marker of vessel-lining endothelial cells, was observed (Fig. 3a,b). Dual staining of CD34, another marker of endothelial cells, with alpha-smooth muscle actin (αSMA) showed that immature or unstable vessels (CD34^+^/αSMA^−^) were primarily affected with bevacizumab treatments, consistent with other published reports^14^ (Fig. 3c and Supplementary Fig. 2). Morphological endothelial cell changes were also evident for the combination treatment, consistent with findings in metastatic melanoma following ipilimumab and bevacizumab treatment^9^. Notably, on-treatment contextual localization of CD68^+^/CD163^+^ but not of CD68^+^/CD163^−^ macrophages was observed adjacent to immature vessels but not mature vessels (Fig. 3c and Supplementary Fig. 3A,B). One potential explanation is that macrophages localized to unstable vessels are responding to bevacizumab-induced vascular and inflammatory effects. Macrophages have been shown to promote vascularization by secreting VEGF, and we found that VEGF transcript expression was upregulated in tumours on-treatment^15^ (Supplementary Fig. 3C).

Intra-tumoral CD8^+^ T-cell increases were pronounced following combination treatment in all but one of the patients (Fig. 3a,b, Supplementary Fig. 4 and Supplementary Table 5). While baseline PD-L1 expression by IHC did not correlate with response to combination therapy, upregulation of PD-L1, an interferon-γ response gene, was only detected by IHC in one patient, who demonstrated a partial response on study (Fig. 3a and Supplementary Table 5). Conversely, we found a concomitant increase in major histocompatibility complex-I (MHC-I) staining with both bevacizumab and combination treatment (Fig. 3a,b). The modulation of MHC-I by anti-VEGF has not been previously described and was not consistently associated with an increase in CD8^+^ T cells. However, studies have found that hypoxia is linked to increased MHC-I expression through hypoxia-inducible factor-1α (ref. 16). Tumour hypoxia by anti-angiogenic therapy stemming from reduction in tumour vasculature may provide one potential mechanism for increase in tumour MHC-I expression and presentation of antigens on the surface of cancer cells.

### Intra-tumoral T-cell repertoires are changed on-treatment

**Figure 4:**
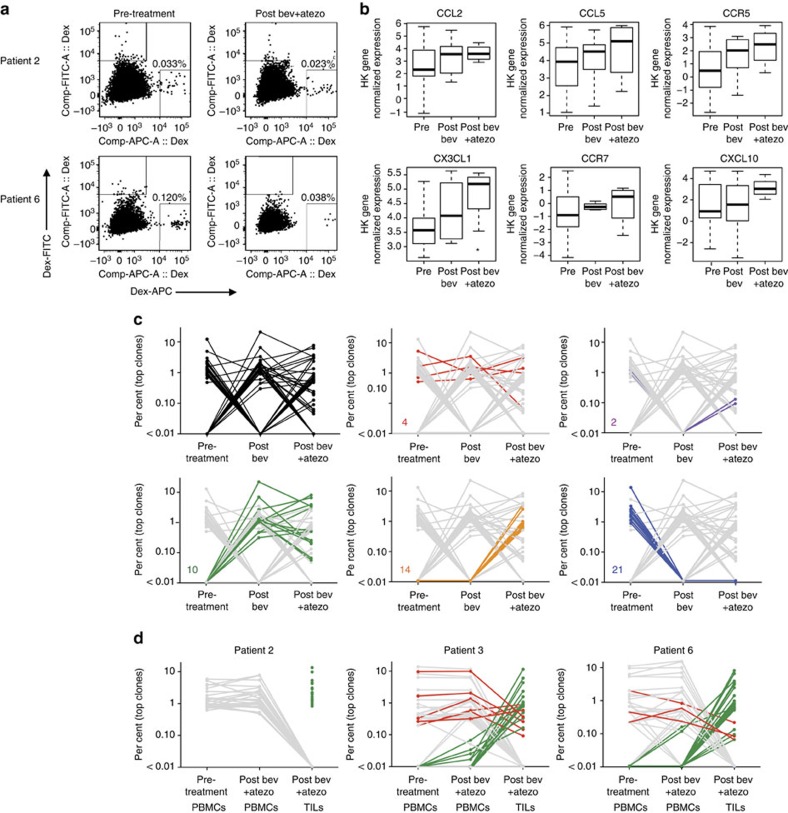
Analyses of antigen-specific T cells in the blood and T-cell repertoire. (**a**) Flow cytometry analyses of antigen-specific T cells (dextramer positive). Representative data from two HLA-A2 positive patients with blood draws matched to tumour biopsy time points are shown. (**b**) Increased expression of chemokines, including CX3CL1 with treatment in tumours. The line in the middle of the box is plotted at the median. Lines above and below the boxes represent variability outside the upper and lower quartiles. (**c**) TCRβ sequencing from patient 6 TILs before and after treatment. The top clones (up to 25) for each group are shown. Prevalence of trending TCRβ clone populations are shown in red, blue, green, orange, and purple. (**d**) TCRβ sequencing of patient 2, 3, and 6 pre-treatment PBMCs, post-bevacizumab (bev) and –atezolizumab (atezo) PBMCs, and post-bevacizumab and -atezolizumab TILs. The top clones (up to 25) for each group are shown.

To address if the increase in CD8^+^ T-cell densities on combination therapy were attributed to enhanced intra-tumoral proliferation or increased trafficking, dual IHC staining of CD8 with the proliferation marker Ki67 was used. The ratio of Ki67^+^/CD8^+^ cells to Ki67^−^/CD8^+^ cells remained unchanged on-treatment (Fig. 3c and Supplementary Fig. 5A,B), suggesting that the increase in CD8^+^ T-cell density was not due to enhanced intra-tumoral proliferation but to increased trafficking and infiltration of proliferating CD8^+^ T cells. This is consistent with the increase in Th1 chemokine signatures associated with bevacizumab treatment. To confirm if the elevation in intra-tumoral CD8^+^ T cells was due to increased trafficking, we phenotyped cell populations in the periphery by flow cytometry. We first utilized human leukocyte antigen (HLA)-A2 dextramers containing previously described RCC tumour antigen peptides to determine if antigen-specific T cells were present in patient blood and if these cell populations changed with treatment^17^,^18^,^19^,^20^ (Supplementary Table 4). Of the 10 patients, 7 were HLA-A2^+^, and dextramer-positive cells could be detected in the Dex-APC channel at the pre-treatment timepoint in 2 patients (patient 2 and 6) (Fig. 4a). Of these 2 patients, only patient 6 demonstrated an increase in intra-tumoral CD8^+^ T cells. Notably, Dex-APC-positive staining decreased more than threefold by the post-combination treatment time point in this patient. While restricted to one observation, these changes suggest that RCC antigen-specific T cells may traffic from the periphery into tumours. Further studies need to be conducted to confirm this observation.

Gene expression data also indicated that several other chemokines and chemokine receptors increased in patient tumours on-treatment (Fig. 4b). The most significant change occurred with fractalkine (CX3CL1), which is known to be expressed on the membrane of activated endothelial cells in inflammatory or hypoxic environments^21^,^22^. The receptor for fractalkine, CX3CR1, is expressed on armed CD8^+^ T cells (perforin^+^/granzyme B^+^)^23^. In the present study, we found that CX3CR1 was upregulated on peripheral CD8^+^ T cells following combination treatment (Supplementary Fig. 6A). Furthermore, the majority of dextramer-positive cells (Fig. 4a) also expressed CX3CR1 (84% and 100% for patients 2 and 6, respectively; Supplementary Fig. 6B). The concordant upregulation of fractalkine and other chemokines in the tumour and CX3CR1 on CD8^+^ T cells on-treatment suggest a mechanism for the increased tumour infiltration of CD8^+^ T cells.

While the increase in Th1-associated chemokine signature points to an association between anti-VEGF treatment and Th1-mediated trafficking of T cells into the tumour, an outstanding question relates to the quality of T-cell response generated by bevacizumab treatment. To address this question, we utilized T-cell receptor (TCR) sequencing on tumours and sorted CD8^+^ T cells from matched peripheral blood mononuclear cells (PBMCs). We attempted to sequence tumour-infiltrating lymphocytes (TILs) from tumours of the six patients, but obtained clone data from pre- and post-treatment biopsies in only two cases due to assay sensitivity. TCR sequencing confirmed an increase in T cells in tumours on-treatment confirming IHC results (Supplementary Table 6). However, the relative frequency of unique clones varied across time points for each patient. Nevertheless, a higher clonality was detected on-treatment for all patients, which may provide evidence for an enhanced tumour-specific response. Comparison of the top 25 clones from pre-treatment and on-treatment TILs for patient 6 showed that while many of the pre-treatment clones were retained in the post-treatment biopsies, anti-VEGF treatment resulted in the emergence of 10 new clones that persisted in combination with atezolizumab, and similar results were obtained for patient 3 TILs (Fig. 4c and Supplementary Fig. 7A). The loss of 21 clones in the post-bevacizumab and combination biopsies highlights one of the challenges with interpreting data from single snapshots in time using TCR technology. This method does not distinguish tumour antigen-specific resident functional T cells from passerby non-functional T cells.

Evaluation of TCR sequences from sorted peripheral CD8^+^ T cells and comparison with tumour CD8^+^ T-cell changes showed no shared clones between PBMCs and TILs for patient 2, the patient where intra-tumoral CD8^+^ T cells did not increase on-treatment. For patients 3 and 6, there were 7 of 25 (28%) and 3 of 25 (12%) of top clones present at similar frequencies between PBMCs and TILs, respectively. However, the majority of top clones in on-treatment TILs were present at much lower levels in the blood while the most dominant clones in the blood are not detected in the tumour. Because the relative proportions of top clones are not maintained in TILs compared with PBMCs, this may suggest that the increase in CD8^+^ T cells in the tumour following combination treatment occurs through a selective trafficking mechanism or there is retention of antigen-specific T cells in the tumour. This is further supported by the observation that viral-specific T-cell clones in the periphery were not detected in the tumour. A blast of the Adaptive Public Clone Database revealed that ∼2.0% (patient 3) and ∼1.4% (patient 6) of all identified PBMC clones may recognize viral antigens^24^. However, none of these clones were detected in patient 3 TILs while <0.3% of patient 6 TILs contained these sequences (Supplementary Fig. 7B). The low frequency of viral antigen-specific T-cell clones in the tumour may provide additional evidence for a T-cell selection process that is preferential to tumour antigens.

## Discussion

The detection and destruction of malignant cells by cytolytic T effector cells is a hallmark of cancer immunotherapy. Thus, targeting pathways that not only enhance the activation but also promote the intra-tumoral trafficking and persistence of tumour-specific T cells has the potential to be a highly effective antitumour strategy. Tumour-secreted VEGF has a dual function in supporting tumour progression: first, by inducing vessel formation and second, by acting as an immunosuppressive factor. In preclinical models tumour growth inhibition following VEGF blockade is associated with increased T-cell numbers within the tumour in addition to reduced vascularity^25^,^26^. Similar results are observed in malignant melanoma patients treated with the combination of bevacizumab and the anti-CTLA4 antibody ipilimumab^9^.

Here we show pharmacodynamic changes and potential mechanisms of action underlying the activity of bevacizumab and the combination of atezolizumab and bevacizumab in mRCC. The combination exhibited a response rate of 40%, which is greater than either single agent alone. Molecular and cellular analyses reveal that bevacizumab treatment alone resulted in decreases in the expected vasculature markers, but also increases in gene signatures associated with Th1 chemokines involved with T-cell trafficking, tumour MHC-I protein expression and infiltration of tumour-specific T-cell clones demonstrating for the first time that bevacizumab is capable of inducing antitumour immune responses. The antitumour activity seen with the combination was associated with a further increase in intra-tumoral CD8^+^ T cells and an increased number of unique T-cell clones in the tumour. This trial was designed as a signal-seeking study to understand the mechanism of bevacizumab-mediated combination activity with atezolizumab in a small number of patients. While we cannot rule out that the biomarker changes over time are driven by bevacizumab treatment alone, enhanced immune-specific responses in combination support a role of PD-L1 blockade. In addition, the consistent directional changes observed for multiple biomarker end points give confidence in interpretation of results from the small cohort of serial biopsies. These results provide evidence for continued evaluation of the atezolizumab and bevacizumab combination in ongoing mRCC phase 2 and phase 3 trials and potential investigation of this combination in other tumour types.

## Methods

### Study oversight

Genentech, Inc., a member of the Roche Group, sponsored the study and supplied the drugs atezolizumab and bevacizumab. Approval for the protocol and its subsequent amendments were obtained from the appropriate institutional review boards or ethics committees. This study was done in compliance with the Declaration of Helsinki and the International Conference on Harmonization Guidelines for Good Clinical Practice. All patients in the study provided a written informed consent. ClinicalTrials.gov: NCT01633970 (https://www.clinicaltrials.gov/ct2/show/NCT01633970?term=GP28328&rank=1).

### Study design

The goal of this study was to evaluate the safety and tolerability of atezolizumab in combination with bevacizumab, a human, monoclonal, engineered anti-VEGF antibody concurrently administered by intravenous infusion every 3 weeks (q3w) to patients with previously untreated advanced mRCC. Treatment was continued as long as patients were experiencing clinical benefit in the opinion of the investigator (that is, in the absence of unacceptable toxicity or symptomatic deterioration attributed to disease progression). Patients were allowed to continue to receive study treatment at the discretion of the investigator if pseudoprogression was suspected or if there was evidence of a mixed response. Study objectives included an evaluation of tumour and circulating pharmacodynamic markers associated with the administration of bevacizumab and atezolizumab and preliminary assessment of the antitumour activity of the treatment combination.

Clinical as well as laboratory safety assessments were done before the start of the treatment and during the course of the trial. The incidence, nature and severity of adverse events were reported according to the National Cancer Institute Common Terminology Criteria for Adverse Events, version 4.0, http://ctep.cancer.gov/protocolDevelopment/electronic_applications/ctc.htm (2013).

Tumours were evaluated at cycles 2, 4, 6, 8, 12 and 16, or as clinically indicated, with the assessments being performed at the end of the 3-week drug-administration cycle and before the beginning of the next cycle. Patients without progressive disease but whose treatment was stopped for other reasons continued to have tumour assessments every 12 weeks until the patient experienced disease progression, initiated further systemic cancer therapy or expired.

Protocol-defined dose limiting toxicity (DLT) criteria included standard grade ≥3 or 4 haematologic and non-haematologic toxicities. Dosing commenced with the recommended phase 2 dose of atezolizumab administered in combination with the labelled q3w dose of bevacizumab, and no DLTs were reported.

### Patients

Patients in this cohort of study GP28328 had advanced or metastatic RCC that did not yet receive systemic therapy, including anti-CTLA-4, anti-PD-1 or anti-PD-L1 therapeutic antibodies, other immunostimulatory or immunosuppressive agents, as well as pathway-targeting agents. Additional eligibility requirements include the following: age ≥18 years old; adequate haematological and end-organ function; Eastern Cooperative Oncology Group performance score of 0 or 1; and measurable disease per RECIST. Patients were excluded if they had known primary central nervous system malignancy or symptomatic central nervous system metastases, and history or risk of autoimmune disease, as well as infection with hepatitis B, hepatitis C or human immunodeficiency virus.

Of the 10 patients on study, 6 yielded biopsies with sufficient viable tumour cells before treatment and at both post-treatment time points. Of the tumours for those 6 patients, 7 were derived from kidney lesions, 4 from the abdominal/chest wall, 1 from a lung lesion, 1 from lymph node and 5 were from undisclosed lesions.

All relevant data are available from the authors.

### Immunohistochemical analysis for PD-L1, CD8 and MHC-I

PD-L1 and CD8 staining were performed as described previously^1^.

All MHC-I IHC steps were carried out on the Ventana Discovery XT automated platform (Ventana Medical Systems, Tucson, AZ). Sections were treated with Cell Conditioner 1, standard time, and then incubated in primary antibody, MHC Class I (EP1395Y, Novus, cat. # NB110-57201) at a 1:5,000 dilution for 60 min at 37 °C. Bound primary antibody was detected by the OmniMap anti-rabbit HRP detection kit, followed by 3,3′-diaminobenzidine (DAB) (Ventana Medical Systems). Sections were counterstained with Hematoxylin II (Ventana Medical Systems) for 4 min, bluing solution for 4 min, then dehydrated and coverslipped. Human cell pellets endogenously expressing low, medium and high MHC-1 were used in parallel as positive controls. Negative controls were performed using rabbit monoclonal (Clone DA1E, Cell Signaling Technology, Catalogue #3900S) isotype antibody. MHC class I staining in tumour cells was scored using an *H*-score system. Briefly, staining intensity of tumour cell membranes was assigned a numerical value of 0, 1, 2 or 3 corresponding to no, low, medium or high DAB signal intensity, respectively. Relative to the overall tumour area, the percentage of cells at different staining intensities was determined by visual assessment. A final score was calculated by multiplying the membrane intensity score by the area percentage for each population present in a given tumour sample: 1 × (% of 1+cells)+2 × (% of 2+cells)+3 × (% of 3+cells)=*H* score. Cases were scored by two independent pathologists. Scoring brackets were defined as scores of ≤100, 101–200 and 201–300, and concordance was defined as independent scores falling within the same bracket. Any discordance was resolved on mutual review of the cases.

### Dual- and triple-colour IHC and a whole-slide digital analysis

Consecutive 4 μm thickness sections of formalin-fixed, paraffin-embedded (FFPE) tumour tissues were stained with the following in-house-developed IHC assays using Ventana Benchmark XT or Benchmark Ultra automated platforms (Ventana Medical Systems): Ki67/CD8, PDPN/CD34/ASMA, CD163/CD68. For Ki67/CD8 assay, sections were treated with Cell Conditioner 1, 64 min, and then incubated in primary antibody, Ki67 (30–9, RTU, Ventana) for 4 min at 37 °C. Bound primary antibody was detected by the OptiView DAB IHC detection kit (Ventana Medical Systems). Subsequently, slides were incubated in primary antibody CD8 (SP239, Spring Biosciences) at a 1:100 dilution for 60 min at 37 °C. Bound primary antibody was detected by the UltraView Universal AP Red detection kit (Ventana Medical Systems). Sections were counterstained with Hematoxylin II (Ventana Medical Systems) for 4 min, bluing solution for 4 min, then dehydrated and coverslipped. For PDPN/CD34/ASMA assay, sections were treated with Cell Conditioner 1, 32 min, and then incubated in primary antibody, Podoplanin (D2-40, RTU, Ventana) for 16 min at 37 °C. Bound primary antibody was detected by the OptiView DAB IHC detection kit (Ventana Medical Systems). Subsequently, slides were incubated in primary antibody CD34 (QBEnd/10; RTU, Ventana) for 16 min at 37 °C. Bound primary antibody was detected by the iView Blue plus detection kit (Ventana Medical System). Finally, slides were incubated in primary antibody SMActin (1A4; RTU, Ventana) for 16 min at 37 °C. Bound primary antibody was detected by the UltraView Universal AP Red detection kit (Ventana Medical Systems). Sections were counterstained with Hematoxylin II (Ventana Medical Systems) for 4 min, bluing solution for 4 min, then dehydrated and coverslipped. For CD163/CD68 assay, sections were treated with Cell Conditioner 1, 32 min, and then incubated in primary antibody, CD163 (MRQ-26, RTU, Ventana) for 8 min at 37 °C. Bound primary antibody was detected by the OptiView DAB IHC detection kit (Ventana Medical Systems). Subsequently, slides were incubated in primary antibody CD68 (KP-1, RTU, Ventana) for 8 min at 37 °C. Bound primary antibody was detected by the UltraView Universal AP Red detection kit (Ventana Medical Systems). Sections were counterstained with Hematoxylin II (Ventana Medical Systems) for 4 min, bluing solution for 4 min, then dehydrated and coverslipped. Appropriate negative and positive controls were performed. Algorithms for the detection and classification of IHC-stained objects on a whole-slide basis were written in Matlab. Following brightfield stain unmixing, IHC-stained objects were detected as cell candidates. For all cell candidates, quantitative features were extracted. Then, candidates were being classified into the various cell classes (for example, CD8^+^/Ki67^−^ cells) using supervised machine learning. The classification method was trained using a ground truth gallery of true- and false-stained objects (provided by a pathologist). Finally, classified cells and tumour areas (provided by a pathologist trough digital slide annotation) were being reported and quality control images being generated for pathology review. The results of automated digital slide analysis were reported for tumour areas as follows: Ki67^−^/CD8^+^ and Ki67^+^/CD8^+^ cell densities (number of cell counts per mm^2^); CD68^+^/CD163^+^ and CD68^+^/CD163^−^ per cent of area coverage (area coverage in relation to the whole tumour area); and CD34^+^/αSMA^−^ and CD34^+^/αSMA^+^ vessel densities (vessel count per mm^2^).

### Nucleic acid isolation from FFPE tumour tissue

Neoplastic tissue was macrodissected from tumour sections and lysed in the presence of Proteinase K to allow for complete digestion and release of nucleic acids. RNA and DNA were isolated using High Pure FFPE RNA Micro Kit (Roche Applied Sciences, Indianapolis, IN) and QIAamp DNA FFPE Tissue Kit (Qiagen, Hilden, Germany), respectively, according to the manufacturer's protocol.

### Fluidigm and Nanostring expression analysis

BioMarkHD real-time PCR (Fluidigm) was performed using either made-to-order or custom-designed FAM-MGB Taqman assays (Life Technologies). Gene expression levels for each sample were calculated with the delta Ct (ΔCt) method using the formula: Ct (target Gene)−Ct (reference genes), where Ct (reference genes) is the geometric median of the Ct values of the four reference genes *SP2*, *GUSB*, *TMEM55B* and *VPS33B*. Patients were divided into high- versus low-expression categories using median mRNA expression levels as cutoffs as measured by immunochip (iChip), and with *P* values determined by *t*-test.

NanoString gene expression data were processed using the R/Bioconductor package ‘NanoStringQCPro' (http://www.bioconductor.org/packages/release/bioc/html/NanoStringQCPro.html). Raw counts were adjusted by positive control counts before probe- and lane-specific background was calculated based on both negative controls and blank measurements. After background correction, counts were log2 transformed and normalized by housekeeping gene expression (*TMEM55B*, *VPS33B*, *TBP* and *TUBB*).

### TCR sequencing

The amplification and sequencing of TCRβ repertoire were performed at Adaptive Biotechnologies as previously described^27^,^28^. Data analyses and the top 25 clones for each group were identified using the ImmunoSEQ Analyzer provided by Adaptive Technologies.

### Flow cytometry

Whole-blood fluorescence-activated cell sorting (FACS) for CD3, CD8, HLA-DR and Ki-67 expression was performed at LabCorp central laboratory according to established protocol. PBMCs were isolated at Precision Bioservices, and cryopreserved samples were shipped to Genentech for analysis of fractalkine receptor expression and detection of tumour-specific T cells. In brief, PBMCs were thawed and rested overnight, then a small aliquot of cells were stained with anti-HLA-A2-FITC (BB7.2, BD) anti-CD45-APC-H7 (2D1, BD) to determine HLA-A2 status. The remaining cells were stained with a mixture of HLA-A*0201/peptide dextramers and pentamer (Immudex and Proimmune, Supplementary Table 4) for 10 min at room temperature followed by anti-CD3-BV510 (UCHTI, Biolegend), anti-CD8-A700 (RPA-T8, BD), anti-CD4-PE-Cy7 (RPA-T4, eBioscience), anti-CD45RA-eVolve605 (HI100, eBioscience), anti-CCR7-BV421 (G043H7, Biolegend), anti-CX3CR1-PerCP-eFluor710 (2A9-1, eBioscience) and Fixable Viability Dye eFluor780 (eBioscience) for 30 min on ice. Samples were washed twice before data acquisition and sorting on a BD FACS Aria running FACSDiva v8 software. A minimum of 10 dextramer-positive events out of 50,000 CD8^+^ T cells is considered a tumour-specific response.

### Statistical analysis

Baseline characteristics and rates of adverse events were obtained using data from all patients (*n*=11) who received at least one dose of atezolizumab and bevacizumab by the clinical cutoff date of 1 September 2015. The efficacy cutoff date is 15 March 2015, with 10 patients being evaluable according to RECIST v1.1. Objective response rate was calculated by dividing the number of patients with a best confirmed overall objective response of complete or partial response by the total number of patients with a baseline tumour assessment. The duration of response was determined through the Kaplan–Meier method, wherein patients who were alive and whose disease did not progress by the cutoff date of 15 June 2015 were censored at the time of last tumour assessment. A summary of all adverse events from the 10 efficacy evaluable patients is provided.

### Data availability

The TCR sequencing data is deposited in the Adaptive Biotechnologies Published Projects Database under the following unique URL: http://adaptivebiotech.com/pub/Wallin-2016-NatureCom. All other data supporting the findings of this study are available within the article and its Supplementary Information Files and from the corresponding author on reasonable request.

## Additional information

**How to cite this article:** Wallin, J. J. *et al*. Atezolizumab in combination with bevacizumab enhances antigen-specific T-cell migration in metastatic renal cell carcinoma. *Nat. Commun.* 7:12624 doi: 10.1038/ncomms12624 (2016).

## Supplementary Material

Supplementary Information Supplementary Figures 1-7 and Supplementary Tables 1-6

Peer Review File Supplementary Information
