# Supplementary material for: Atezolizumab in combination with bevacizumab enhances antigen-specific T-cell migration in metastatic renal cell carcinoma
Source: Nat Commun. 2016 Aug 30;7:12624. doi: 10.1038/ncomms12624 (PMC5013615; doi:10.1038/ncomms12624)
Supplement: Supplementary Information — Supplementary Figures 1-7 and Supplementary Tables 1-6 [file ncomms12624-s1.pdf]

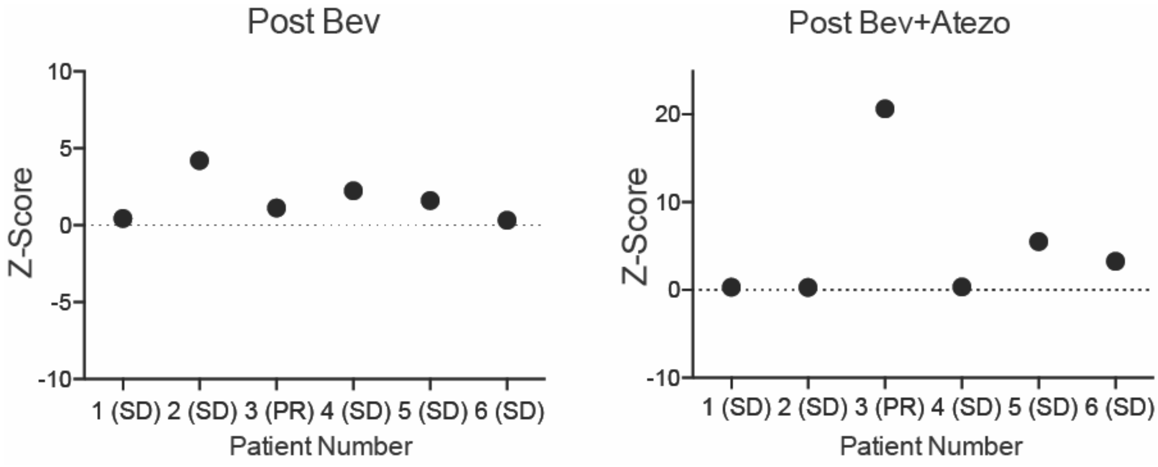

Figure 1. FASLG gene expression following treatments. FASLG expression levels of on-treatment tumor samples (black dots) are shown relative to the baseline levels (dotted line) for the patients where the pre-treatment, post bevacizumab and post combination biopsies were collected.

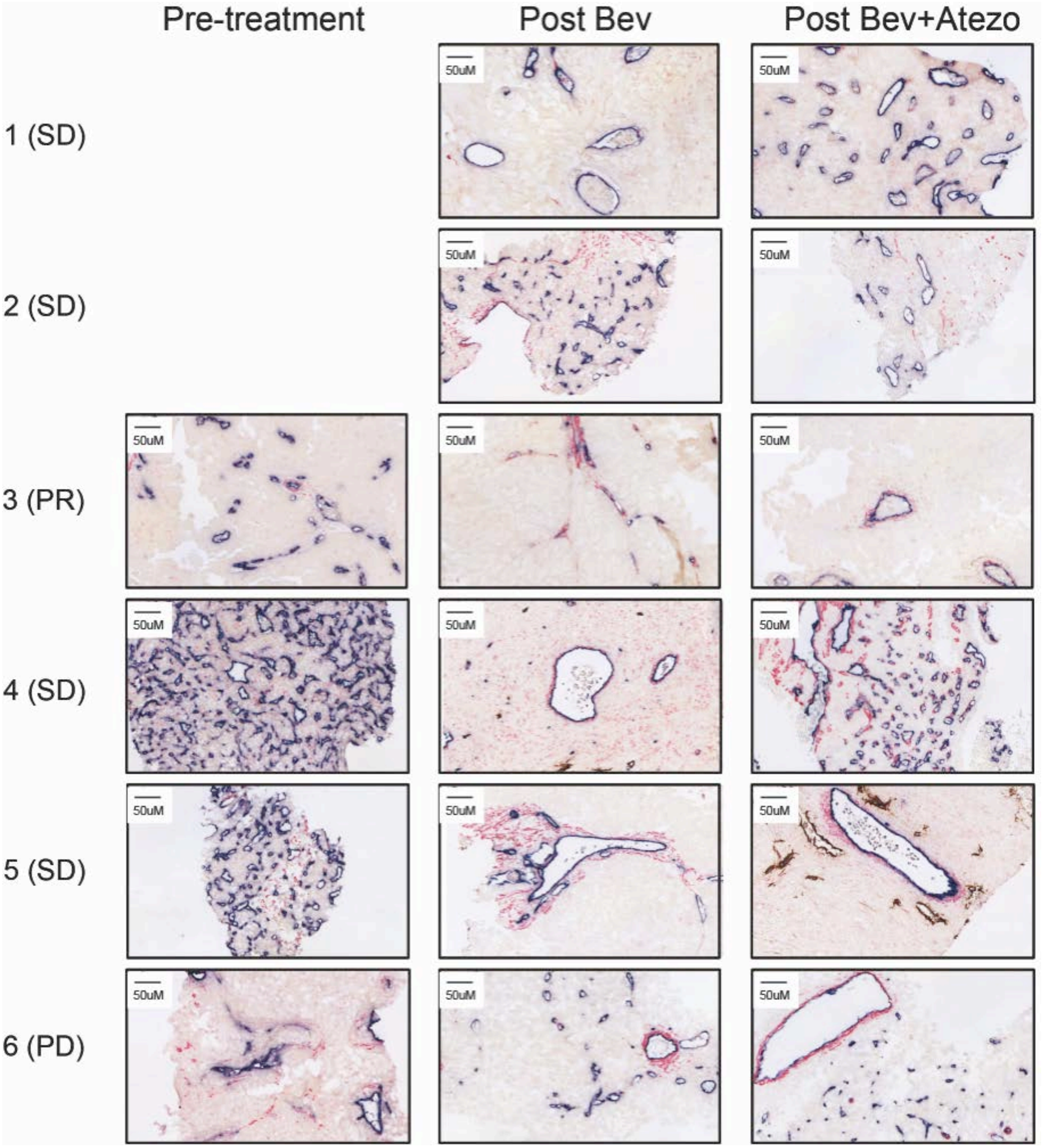

Figure 2. Vasculature markers in baseline and on-treatment tumor samples. Representative images of CD34 (blue), αSMA (red), and podoplanin (brown) from patient 1-6 tumor samples. Bev, bevacizumab; Atezo, atezolizumab; SD, stable disease; PR, partial response; PD, progressive disease. A scale bar for each image representing 50um is shown.

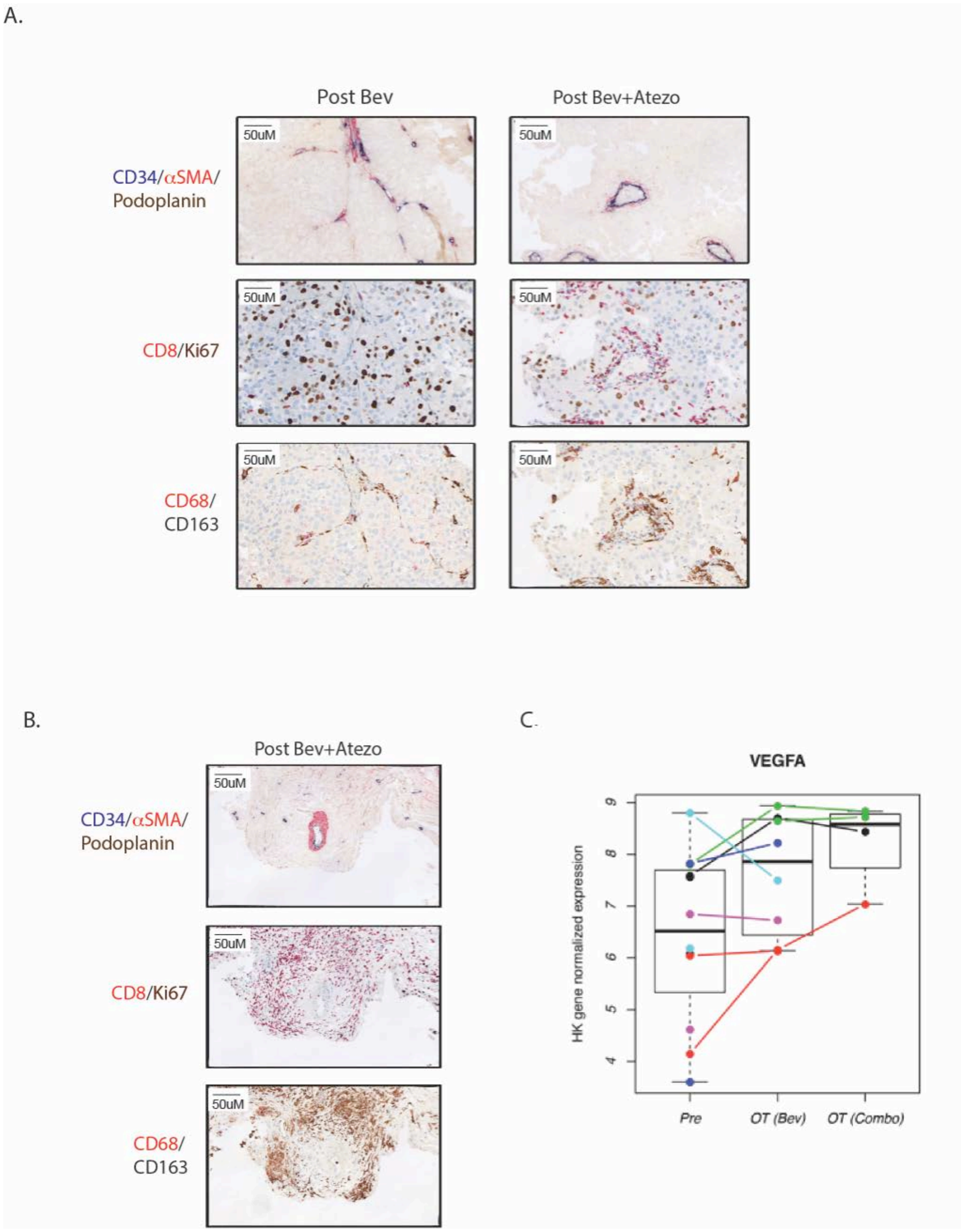

Figure 3. Protein expression of immune/vasculature markers and VEGFA gene expression in baseline and on-treatment tumor samples. a and b, Representative immunofluorescence images of serial sections around immature (a) or a mature (b) vessel(s) from patient 3 tumors. Top images are stained for vascular markers - CD34 (blue), αSMA (red), and podoplanin (brown), middle images are stained for CD8+ T-cells - CD8 (red) and Ki67 (brown), and bottom images are stained for macrophages - CD68 (red) and CD163 (brown). c, VEGFA gene expression from pre-treatment, post bevacizumab (OT (Bev)) and post bevacizumab in combination with atezolizumab (OT (Combo)). Connecting lines are data collected for the same patient across more than one timepoint. A scale bar for each image representing 50um is shown.

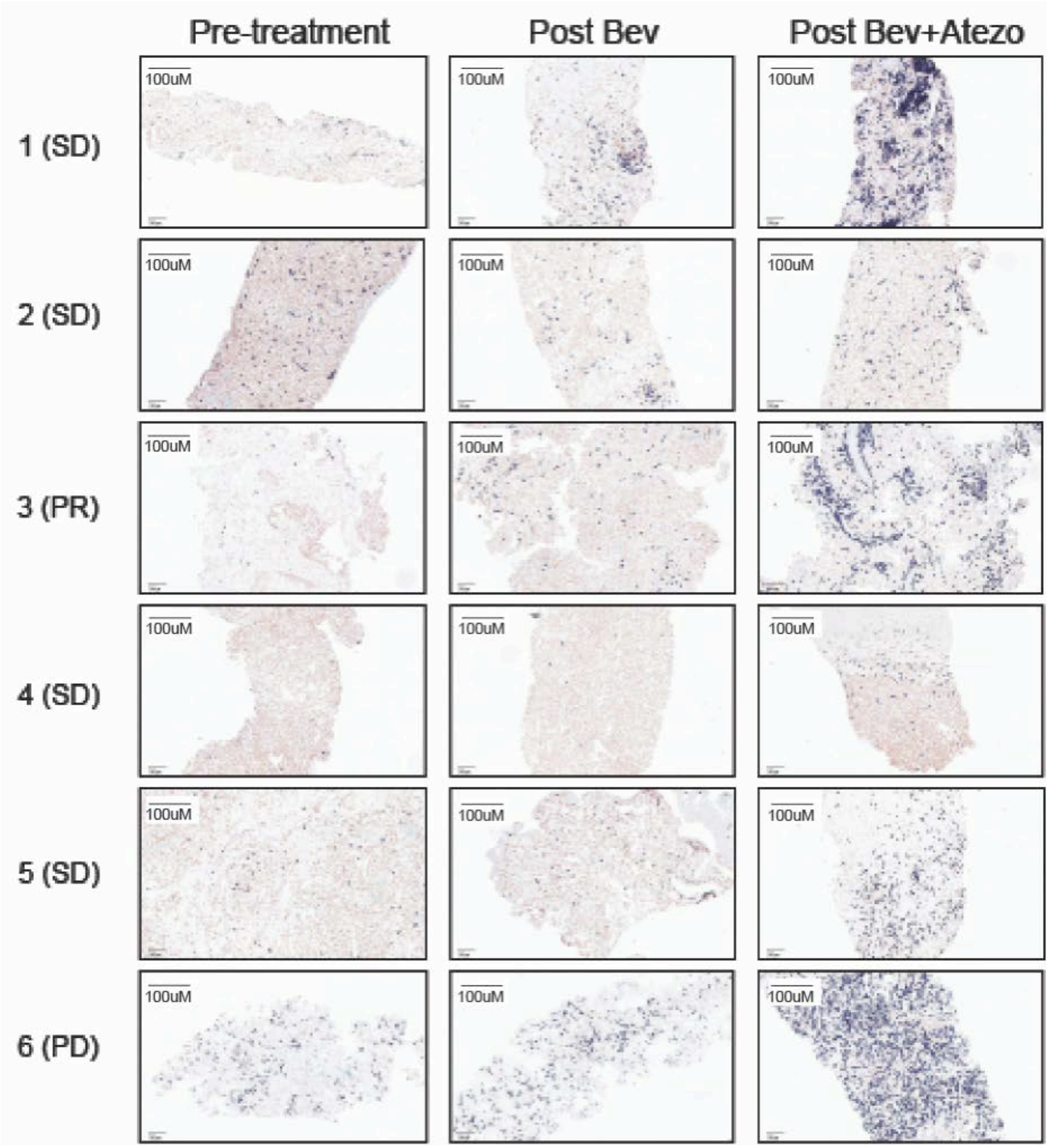

Figure 4. Increases in $CD8^+$ T cells at the post bev+atezo combination timepoint. Representative CD8 IHC images from pre-treatment and on-treatment patient tumor samples. $CD8^+$ T cells are stained in blue. Bev, bevacizumab; Atezo, atezolizumab; SD, stable disease; PR, partial response; PD, progressive disease. A scale bar for each image representing 100um is shown.

A.

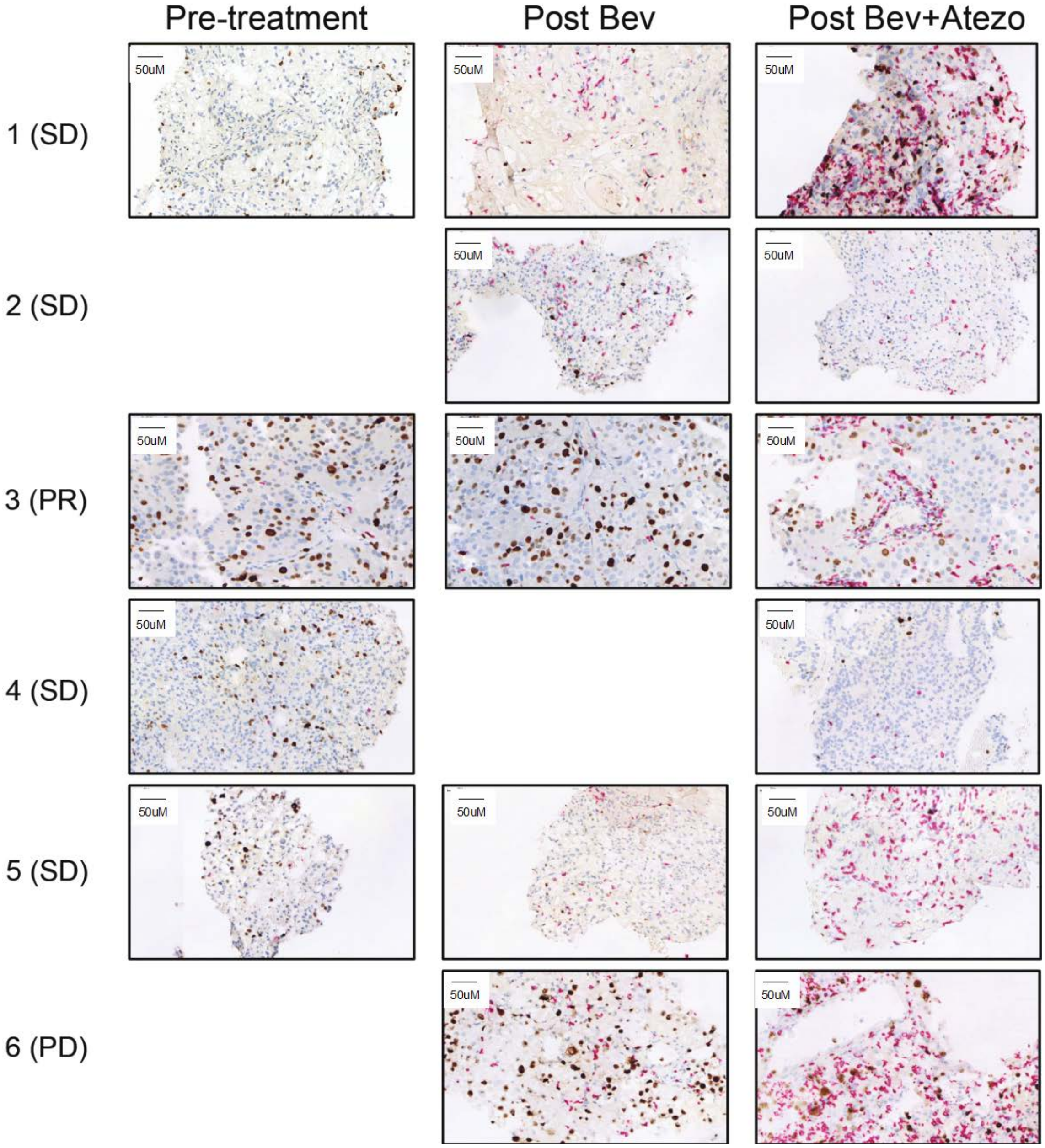
Pre-treatment
Post Bev
Post Bev+Atezo
1 (SD)
2 (SD)
3 (PR)
4 (SD)
5 (SD)
6 (PD)
50uM

B.

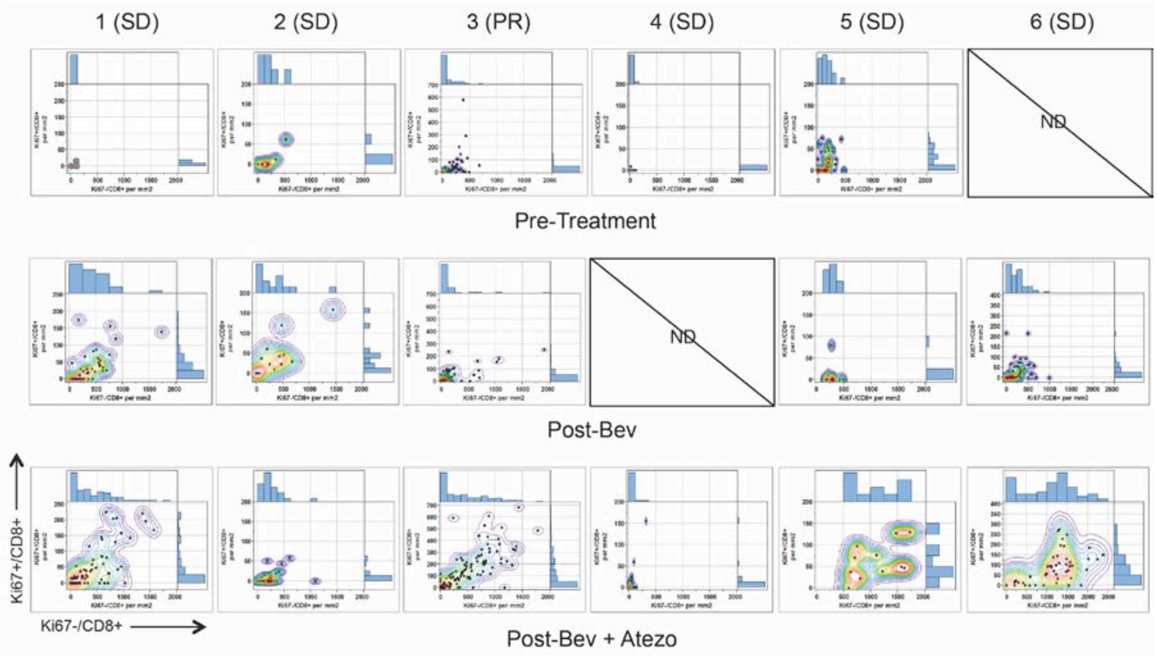

Figure 5. Staining for proliferating $CD8^+$ T cells ($Ki67^+$) in pre-treatment and on-treatment tumors. a, Representative CD8 (red) and Ki67 (brown) immunofluorescence images from pre-treatment and on-treatment patient tumor samples. Bev, bevacizumab; Atezo, atezolizumab; SD, stable disease; PR, partial response; PD, progressive disease. A scale bar for each image representing 50um is shown. b, quantification of $CD8^+/Ki67^-$ and $CD8^+/Ki67^+$ staining from immunofluorescence images. Proliferating ($Ki67^+/CD8^+$) T cells are shown on the Y-axis and non-proliferating $CD8^+$ T cells ($Ki67^-/CD8^+$) on the x-axis. Each dot represents quantification of $CD8^+/Ki67^-$ and $CD8^+/Ki67^+$ cells per mm2.

A.

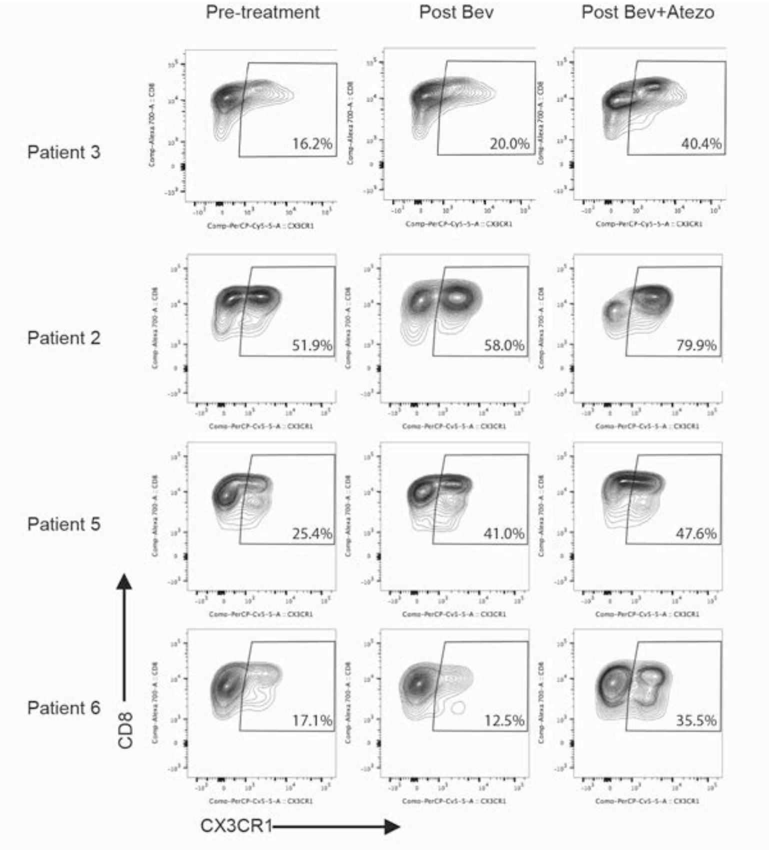

B.

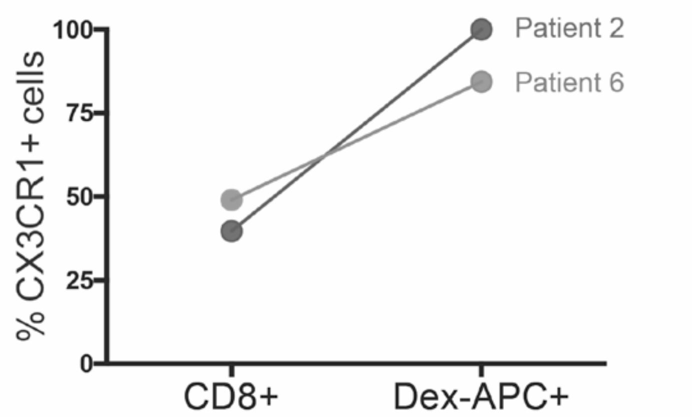

Figure 6. Increases in CX3CR1 expression on-treatment for CD8^{+} T-cells. a, flow cytometry staining of CX3CR1 staining on CD8^{+} T-cells from patients 2, 3, 5, and 6. b, comparison of CX3CR1 positivity pre-treatment in dextramer negative (CD8^{+}) and dextramer positive (Dex-APC^{+}) CD8^{+} cells for patients 2 and 6.

A.

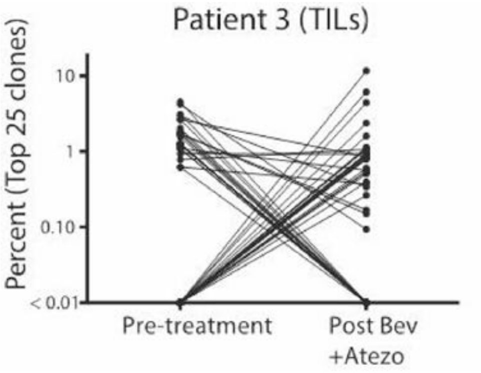

B.

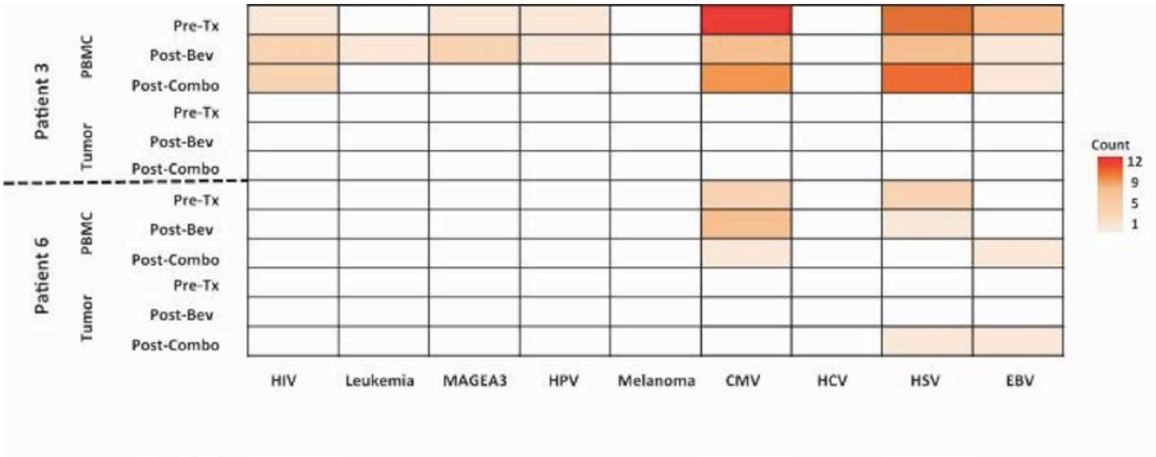

Figure 7. Changes in patient T cell receptor clones in on-treatment PBMCs and TILs. a, TCRβ sequencing from patient 3 TILs before and after treatment. The top clones (up to 25) for each group are shown. b, prevalence of TCRβ sequences of known specificities from the Adaptive Public Clone Database in pre-treatment and on-treatment PBMC and TIL samples from patients 3 and 6.

Table 1. Baseline Demographics

Characteristics	N = 11
Median age (range), y	59 (42-74)
Male, n (%)	8 (73%)
Median safety follow-up month (range)	17.2 (2.3-20.4)
ECOG PS 1, %	3 (27%)
Prior systemic therapy	0 (0%)

- Study doses were confirmed as 20 mg/kg q3w of MPDL3280A and 15 mg/kg q3w bevacizumab
- Median duration of treatment for MDPL3280A was 15.9 months (range: 1 to 19 months)
- Overall, treatments were well tolerated with fatigue, decreased appetite, arthralgia, and nausea being the most common adverse events (AEs) (Tables 2-3)
    - -6 Grade 3-4 AEs regardless of attribution occurred (Table 2)
    - No Grade 5 AEs regardless of attribution were reported
    - No Grade 3-4 AEs deemed possibly related to MPDL3280A per investigator were observed (Table 3)

Table 2. AEs Occurring in >= 4 Patients Regardless of Attribution

AEs, n (%)	All Grade[a] N = 11	Grade 3-4[b] N = 11
All	11 (100%)	6 (54.5%)
Fatigue	10 (91%)	0
Nausea	6 (54.5%)	0
Decreased appetite	5 (45.5%)	0
Pruritus	5 (45.5%)	0
Pyrexia	4 (36%)	0
Pain in extremity	4 (36%)	0
Hypertension	4 (36%)	3 (27%)
Dyspnoea	4 (36%)	0
Epistaxis	4 (36%)	0
Productive cough	4 (36%)	0

[a] AEs ≥ 4 patients.
[b] Additional Grade 3-4 AEs included acute respiratory failure (10%), hypercalcemia (10%), and abdominal pain (10%).

Table 3. AEs Related to MPDL3280A Occurring in >=2 Patients

AEs[a], n (%)	N = 11
All	9 (82%)
Fatigue	7 (82%)
Chills	3 (27%)
Decreased appetite	3 (27%)
Diarrhea	3 (27%)
Rash	2 (18%)
Nausea	2 (18%)
Vomiting	2 (18%)
Pruritus	2 (18%)

[a] All AEs are Grade 1 or 2.
[b] AE reported term is used for uncoded events.

Table 4. Dextramers for FACS

RCC-specific/associated antigens		
**Dex-FITC**	**Dex-PE**	**Dex-APC**
APOL1	MAGE-A1	G250 217-225
APOL1	PRAME-1	NY-ESO-1
MUC-1 12-20	PRAME-2	PRAME-4
MUC-1 13-21	PRAME-3	PRAME-5
SSX-2	Survivin	PRAME-6
SSX-2	CCND1	Survivin
DLK1	GUCY1A3*	MET
EphA2	Hsp70-2*	PLIN
NRP1	IDO*	PRUNE2
PDGFRβ*	FLT1	RGS5*
TEM1	KDR	

Table 5. CD8 and PDL1 Quantification in Patient Tumors

PATIENT ID	Visit	% Total Infiltrate	CD8 Central Tumor	PDL1 IC RAW	PDL1 TC RAW	PDL1 IC score
1	Pre	10	0.71	0	0	0
	Post-Bev	10	2.62	<1	0	0
	Post-Combo	15	6.98	10	0	3
2	Pre	<1	1.06	0	0	0
	Post-Bev	5	1.38	<1	0	0
	Post-Combo	2	1.37	<1	0	0
3	Pre	15	0.24	<1	0	0
	Post-Bev	15	1.17	1	0	1
	Post-Combo	20	4.63	10	40	3
4	Pre	1	0.06	0	0	0
	Post-Bev	3	0.11	0	0	0
	Post-Combo	2	0.33	0	0	0
5	Pre	1	1.07	0	0	0
	Post-Bev	10	1.2	0	0	0
	Post-Combo	NA	3.72	NA	NA	NA
6	Pre	1	1.9	<1	100	0
	Post-Bev	15	1.56	1	100	1
	Post-Combo	30	5.37	10	100	3
7	Pre	20	3.5	20	0	3
	Post-Bev	NA	NA	NA	NA	NA
	Post-Combo	NA	NA	NA	NA	NA
8	Pre	NA	NA	NA	NA	NA
	Post-Bev	NA	NA	NA	NA	NA
	Post-Combo	NA	NA	NA	NA	NA
9	Pre	30	7.53	1	5	1
	Post-Bev	4	4.54	1	2	1
	Post-Combo	NA	NA	NA	NA	NA
10	Pre	1	0.98	0	0	0
	Post-Bev	10	1.75	2	0	1
	Post-Combo	NA	NA	NA	NA	NA

Table 6. TCR Sequencing Data from Patient 2, 3, and 6 Tumors

Samply Type	Patient No.	Visit	T cell fraction	Total Productive	Unique Productive	% Unique	Frequency of Top Clone	Clonality
TILs	2	Pre	NA	NA	NA	NA	NA	NA
	2	Post-Bev	0.07	98	2	2.04	41.98	0.11
	2	Post-Combo	0.17	1175	51	4.34	12.83	0.13
	3	Pre	0.08	553	84	15.19	4.52	0.05
	3	Post-Bev	NA	NA	NA	NA	NA	NA
	3	Post-Combo	0.36	4411	249	5.64	11.26	0.17
	6	Pre	0.06	466	8	1.72	30.27	0.11
	6	Post-Bev	0.09	2174	75	3.45	21.06	0.20
	6	Post-Combo	0.31	9519	355	3.73	7.64	0.18
PBMC	2	Pre	NA	1484	297	20.01	5.42	0.18
	2	Post-Bev	NA	785	200	25.48	6.01	0.17
	2	Post-Combo	NA	4912	497	10.12	7.23	0.27
	3	Pre	NA	1266	439	34.68	13.90	0.29
	3	Post-Bev	NA	2411	1030	42.72	9.54	0.24
	3	Post-Combo	NA	10105	2367	23.42	11.73	0.37
	6	Pre	NA	310	103	33.23	10.40	0.24
	6	Post-Bev	NA	1703	370	21.73	15.45	0.35
	6	Post-Combo	NA	678	119	17.55	14.98	0.35
