# Supplementary material for: Atezolizumab in combination with bevacizumab enhances antigen-specific T-cell migration in metastatic renal cell carcinoma
Source: Nat Commun. 2016 Aug 30;7:12624. doi: 10.1038/ncomms12624 (PMC5013615; doi:10.1038/ncomms12624)
Supplement: Peer Review File — Supplementary Information [file ncomms12624-s2.pdf]

Reviewers' comments:

Reviewer #1 (Immunotherapy expert) (Remarks to the Author):

A. Summary of the key results
This study elegantly demonstrates the sequential effects of bevacizumab followed by bevacizumab and atezolizumab on biomarker expression in renal tumours. The difficulty of obtaining sequential tissues should not be underestimated, justifying the small numbers. The trial design is excellent allowing us to unpick the effects of VEGF targeted therapy and PD-L1 targeted therapy on the tumor. The authors deserve congratulation.

B. Originality and interest: if not novel, please give references
This work is ahead of its time. The difficulty of collecting sequential tissue should not be underestimated. The immunogenic effects of bevacizumab are novel. All of the combination data is novel. Its important because the combination is in randomised phase III trials and may change the treatment of renal cancer. If the randomised trials are positive this translational work will be cited as biomarker data supporting the use of the combination. The quality of the science and the impact of the results justifies the publication in a high impact journal.

C. Data & methodology: validity of approach, quality of data, quality of presentation
Excellent.

D. Appropriate use of statistics and treatment of uncertainties
I would be tempted to remove all response rate data and reference to comparison with response to other agents. The numbers are so small. The fact than one patient responded at a particular time with single biomarker expression may be overcalling the data.

E. Conclusions: robustness, validity, reliability
Excellent.

F. Suggested improvements: experiments, data for possible revision
Figure 4 on T cell clones required better explanation in the text. The small numbers available mean this data is hard to interpret. The authors should outline what they were trying to achieve and what they were able to interpret from the limited data available. Is there a better way of presenting this data. The inconsistency between the results in the blood and those intratumourally are particularly interesting. They suggest a process of selection within the tumor. Does this throw up flaws for future circulating biomarker research.

It is likely that time dependant changes occur with therapy. It is therefore conceivable that changes seen with the addition of the 2nddrugs are not related to the effects of atezolizumab but an evolution of the tumour reaction to bev related hypoxia. Immune specific responses, seen in the manuscript argue against this. Could the authors add a section to address this? It should also be cited as a potential shortcoming.

A discussion about the potential antagonistic or synergistic interaction bases on these data should be discussed to guide future trials in this area. One would imagine that the CD8 infiltration after Atezo would support the latter.

G. References: appropriate credit to previous work?
Yes
H. Clarity and context: lucidity of abstract/summary, appropriateness of abstract, introduction and conclusions
Excellent.

Reviewer #2 (Kidney cance, immunotherapy expert)(Remarks to the Author):

The manuscript "Opening the floodgates to T-cells: clinical activity and immune correlates from a phase Ib study evaluating atezolizumab (anti-PDL1) in combination with bevacizumab (anti-VEGF) in metastatic renal cell carcinoma" by Wallin et al reports an early phase trial combining bevacizumab and atezolizumab in a small number of patients. Patients underwent multiple biopsies on study with the purpose to assess the mechanisms of tumor response. The combination resulted in a 40% response proportion in 10 evaluable patients. The authors report an increase in intra-tumoral CD8 positive T-cells, intra-tumoral MHC-I, and increases in CX3CL1, suggesting anti-VEGF and anti-PD-L1 improves the migration of antigen-specific T-cells into the renal tumors. The sequential biopsy of patients is not novel but informative, as is many of the assays used in the study.
The major deficiency of this study is the small sample size. Frequently data is only available for interpretation from very few patients (<6) and the authors are left speculating on interpretation of the data. This study is informative but speculative, and likely has many results that will be validated. Other corroborating data (perhaps from a pre-clinical model) would benefit the interpretation.
Why was a higher dose of 15 mg/kg q 3 weeks of bevacizumab used in this study? Hypertension was only seen in 4 patients. Treatment related hypertension is a predictor of response to anti-VEGF therapy. Was hypertension seen in the responding patients? The response proportions and duration is high but may not be statistically different then what would be expected in this small cohort of patients. It is unclear if this cohort of patients is more likely to respond to an PDL-1 inhibitor. The authors described PDL-1 staining in the methods but do not specifically show the percent and how this changed with time. Did any of the patients have no immune infiltrate at baseline? A table outlining some of the suspected predictors of response to PDL-1 inhibition in each pt over time would be useful.
The figure legends in the supplement should be expanded to assist the reader in the interpretation of the figures.
Minor comments: The word tissue is repeated twice in the abstract.

Reviewer #3 (sequencing expert) (Remarks to the Author):

Summary of the key results
Originality and interest: if not novel, please give references

Study appears original, interesting and valuable

Data & methodology: validity of approach, quality of data, quality of presentation

The sequencing results and conclusions are highly dependent on the volume of data, sample size and sample quality. The study is difficult to evaluate given that no information on the sequencing data was presented.

Appropriate use of statistics and treatment of uncertainties
The authors suggest a role for tumor antigen specificity in the infiltrating T cells however evidence still needs to be presented to argue against what I would consider the null-hypothesis that the tumor repertoire post treatment is non-specific, from the periphery and that variability is due to sampling effects.

Conclusions: robustness, validity, reliability

TCR sequencing is robust and well validated but FFPE samples often produce poor data due to the nature of the material. A table of number of uniques, number of T cells, frequency of the top clone and clonality would aid interpretation.

Suggested improvements: experiments, data for possible revision
A table of the sequencing data. Additional explanation of how they counted presence/absence of clones in the top 25 and how that fits in with overlap and correlation between tumor and PBMC and across time points would help evaluate their conclusions.

References: appropriate credit to previous work?

Done.

Clarity and context: lucidity of abstract/summary, appropriateness of abstract, introduction and conclusions

The paper is well written, easy to read and understand. I think the sequencing results and discussion would be greatly enhanced by additional review. I think stronger conclusions could be made based on additional statistical analysis. Specifically whether the patterns they see regarding overlap across time points and between the PBMC and the tumor can be explained by sampling alone.

Reviewer #1 (Immunotherapy expert) (Remarks to the Author):

A. Summary of the key results
This study elegantly demonstrates the sequential effects of bevacizumab followed by bevacizumab and atezolizumab on biomarker expression in renal tumours. The difficulty of obtaining sequential tissues should not be underestimated, justifying the small numbers. The trial design is excellent allowing us to unpick the effects of VEGF targeted therapy and PD-L1 targeted therapy on the tumor. The authors deserve congratulation. We thank the reviewer for their supportive comments.

B. Originality and interest: if not novel, please give references
This work is ahead of its time. The difficulty of collecting sequential tissue should not be underestimated. The immunogenic effects of bevacizumab are novel. All of the combination data is novel. Its important because the combination is in randomised phase III trials and may change the treatment of renal cancer. If the randomised trials are positive this translational work will be cited as biomarker data supporting the use of the combination. The quality of the science and the impact of the results justifies the publication in a high impact journal. We thank the reviewer for their supportive comments.

C. Data & methodology: validity of approach, quality of data, quality of presentation
Excellent.

D. Appropriate use of statistics and treatment of uncertainties
I would be tempted to remove all response rate data and reference to comparison with response to other agents. The numbers are so small. The fact than one patient responded at a particular time with single biomarker expression may be overcalling the data. We agree that these are small numbers, but feel the response data is needed for background to the study. Due to the small numbers, we do not distinguish biomarker data based on response data in the manuscript.

E. Conclusions: robustness, validity, reliability
Excellent.

F. Suggested improvements: experiments, data for possible revision
Figure 4 on T cell clones required better explanation in the text. The small numbers available mean this data is hard to interpret. The authors should outline what they were trying to achieve and what they were able to interpret from the limited data available. Is there a better way of presenting this data. The inconsistency between the results in the blood and those intratumourally are particularly interesting. They suggest a process of selection within the tumor. Does this throw up flaws for future circulating biomarker research. Thank you for this comment. We added more background and interpretation for this data in the text.

It is likely that time dependant changes occur with therapy. It is therefore conceivable that changes seen with the addition of the 2nddrugs are not related to the effects of atezolizumab but an evolution of the tumour reaction to bev related hypoxia. Immune specific responses, seen in the manuscript argue against this. Could the authors add a section to address this? It should also be cited as a potential shortcoming. We agree with this comment and added text in the final paragraph to bring this point to the attention of the reader.

G. References: appropriate credit to previous work?
Yes

H. Clarity and context: lucidity of abstract/summary, appropriateness of abstract, introduction and conclusions
Excellent.

Reviewer #2 (Kidney cancer, immunotherapy expert) (Remarks to the Author):

The manuscript "Opening the floodgates to T-cells: clinical activity and immune correlates from a phase Ib study evaluating atezolizumab (anti-PDL1) in combination with bevacizumab (anti-VEGF) in metastatic renal cell carcinoma" by Wallin et al reports an early phase trial combining bevacizumab and atezolizumab in a small number of patients. Patients underwent multiple biopsies on study with the purpose to assess the mechanisms of tumor response. The combination resulted in a 40% response proportion in 10 evaluable patients. The authors report an increase in intra-tumoral CD8 positive T-cells, intra-tumoral MHC-I, and increases in CX3CL1, suggesting anti-VEGF and anti-PD-L1 improves the migration of antigen-specific T-cells into the renal tumors. The sequential biopsy of patients is not novel but informative, as is many of the assays used in the study.
The major deficiency of this study is the small sample size. Frequently data is only available for interpretation from very few patients (<6) and the authors are left speculating on interpretation of the data. This study is informative but speculative, and likely has many results that will be validated. Other corroborating data (perhaps from a pre-clinical model) would benefit the interpretation.

Why was a higher dose of 15 mg/kg q 3 weeks of bevacizumab used in this study? In the larger pan-tumor Phase 1b study we wanted to test the safety and tolerability of the atezo + bev combination with application to as many indications as possible. We determined that there was no need to lower the dose specifically for the RCC cohort as we used the approved dose level (bev is approved at 10 mg/kg q2w).

Hypertension was only seen in 4 patients. Treatment related hypertension is a predictor of response to anti-VEGF therapy. Was hypertension seen in the responding patients? Thank you for this question. Three out of the four patients with partial responses had hypertension that was deemed related to bev. This association will be further investigated in the phase 2 study.

The response proportions and duration is high but may not be statistically different then what would be expected in this small cohort of patients. It is unclear if this cohort of patients is more likely to respond to an PDL-1 inhibitor. The authors described PDL-1 staining in the methods but do not specifically show the percent and how this changed with time. Did any of the patients have no immune infiltrate at baseline? A table outlining some of the suspected predictors of response to PDL-1 inhibition in each pt over time would be useful. Yes - some of the patients had very low levels of infiltrates at baseline. External data table 5 has been added which displays %total infiltrate, CD8 in the central tumor and PDL1 data.

The figure legends in the supplement should be expanded to assist the reader in the interpretation of the figures. The figure legends have been expanded.

Minor comments: The word tissue is repeated twice in the abstract. Thanks for noticing this. It has been corrected.

Reviewer #3 (sequencing expert) (Remarks to the Author):

Summary of the key results
Originality and interest: if not novel, please give references

Study appears original, interesting and valuable

Data & methodology: validity of approach, quality of data, quality of presentation

The sequencing results and conclusions are highly dependent on the volume of data, sample size and sample quality. The study is difficult to evaluate given that no information on the sequencing data was presented.

Appropriate use of statistics and treatment of uncertainties
The authors suggest a role for tumor antigen specificity in the infiltrating T cells however evidence still needs to be presented to argue against what I would consider the null-hypothesis that the tumor repertoire post treatment is non-specific, from the periphery and that variability is due to sampling effects. In this study we evaluated multiple biomarker endpoints and the consistency of these readouts gives us confidence in these endpoints.

Conclusions: robustness, validity, reliability
TCR sequencing is robust and well validated but FFPE samples often produce poor data due to the nature of the material. A table of number of uniques, number of T cells, frequency of the top clone and clonality would aid interpretation. Thank you for this suggestion. A table has been added (External Data Table 6) that contains this information.

Suggested improvements: experiments, data for possible revision
A table of the sequencing data. Additional explanation of how they counted presence/absence of clones in the top 25 and how that fits in with overlap and correlation between tumor and PBMC and across time points would help evaluate their conclusions. A data table has been added and additional explanation of the TCR sequencing data is now included in the text.

References: appropriate credit to previous work?

Done.

Clarity and context: lucidity of abstract/summary, appropriateness of abstract, introduction and conclusions

The paper is well written, easy to read and understand. I think the sequencing results and discussion would be greatly enhanced by additional review. I think stronger conclusions could be made based on additional statistical analysis. Specifically whether the patterns they see regarding overlap across time points and between the PBMC and the tumor can be explained by sampling alone.

REVIEWERS' COMMENTS:

Reviewer #1 (Remarks to the Author):

I think this is improved, especially the discussion. I think it should be published.
I would still recommend removing the response data.

Reviewer #2 (Remarks to the Author):

The authors have addressed the majority of the reviewer's concerns. The major deficiency of the manuscript remains, which is the small sample size. The study is provocative and provides rationale to study more patients treated with this regimen.

Reviewer #3 (Remarks to the Author):

In regards to the author response to reviewer #3 suggestions.

The table helps enormously in reviewing the sequencing results. It's challenging to interpret samples that produce poor data. All the TIL data was marginal for measuring clonality, % T cells and changes in total T cells (particularly patient 2) and Patient 6 PBMC results were also surprisingly poor. However the authors study utilized multiple independent lines of evidence and the sequencing results either corroborated their conclusions or remained silent. Reviewing the table, I agree with their conclusion, that the intra-tumoral environment clearly increased in T cells and remained or became more polyclonal. I do not find the data sufficient to conclude there is a unique tumor repertoire simply because the sample sizes are small enough that the amount of overlap and correlation of shared clones is going to be dominated by sampling effects. It is challenging to interpret these metrics in particular with smaller samples.

I recommend publication at this time.

Reviewer #1 (Remarks to the Author):
I think this is improved, especially the discussion. I think it should be published.
I would still recommend removing the response data. We think that the response data provides context to the study and is something that readers will want to know. Response data has been removed from the updated abstract.

Reviewer #2 (Remarks to the Author):
The authors have addressed the majority of the reviewer's concerns. The major deficiency of the manuscript remains, which is the small sample size. The study is provocative and provides rationale to study more patients treated with this regimen. Thank you for these comments. We agree that this is a small sample size, but believe this cohort of patient data is unique due to the success of biopsy collection.

Reviewer #3 (Remarks to the Author):
In regards to the author response to reviewer #3 suggestions.
The table helps enormously in reviewing the sequencing results. It's challenging to interpret samples that produce poor data. All the TIL data was marginal for measuring clonality, % T cells and changes in total T cells (particularly patient 2) and Patient 6 PBMC results were also surprisingly poor. However the authors study utilized multiple independent lines of evidence and the sequencing results either corroborated their conclusions or remained silent. Reviewing the table, I agree with their conclusion, that the intra-tumoral environment clearly increased in T cells and remained or became more polyclonal. I do not find the data sufficient to conclude there is a unique tumor repertoire simply because the sample sizes are small enough that the amount of overlap and correlation of shared clones is going to be dominated by sampling effects. It is challenging to interpret these metrics in particular with smaller samples. Thank you for these comments. We agree that the supplemental tables aid interpretation of the data.

I recommend publication at this time.
